# Rethinking culture: a narrative review on the evolving role of museum and art gallery-based heritage activities and programmes on wellbeing

**DOI:** 10.1177/17579139241268446

**Published:** 2024-09-27

**Authors:** J Fares, I Hadjicosti, C Constantinou

**Affiliations:** University of Nicosia Medical School, Cyprus; Bank of Cyprus Cultural Foundation, Cyprus; Deputy Ministry of Culture, Cyprus; Department of Basic and Clinical Sciences, University of Nicosia Medical School, 21 Ilia Papakyriakou, 2414 Engomi, P.O. Box 24005, CY-1700 Nicosia, Cyprus

**Keywords:** museums, art gallery-based programmes, addiction recovery, dementia, wellbeing, younger, older

## Abstract

**Aims:**

The prevalence of chronic mental and physical diseases is increasing globally. In addition, the changing demographics towards an ageing population pose a challenge to healthcare systems, as ageing is associated with a decrease in physical and mental capacity and an increased risk of developing disease. The review aims to explore primary studies that investigated the effect of museum and art gallery-based heritage activities and programmes on the wellbeing of (1) individuals recovering from drug addiction or patients with dementia and (2) younger and older adults.

**Methods:**

We conducted a search using specific keywords, and inclusion and exclusion criteria, in databases for the period 2013–2023. Following a detailed examination of numerous articles, 15 original studies were included in this review.

**Results:**

15 original studies investigated the effects of museum and art gallery-based heritage activities or programmes on (1) patients with chronic diagnoses associated with mental health and/or physical impairment, such as drug addiction and dementia and (2) the wellbeing of younger and older populations. The interactive environment of museums had positive health outcomes for patients with chronic mental (addiction recovery, dementia) and physical diseases (cancer) as well as hospitalized patients. In addition, it improved the physical and mental wellbeing of younger and older individuals.

**Conclusion:**

Museum art-based interventions may be integrated as part of the non-pharmacological management of patients experiencing mental disorders as well as for improving the wellbeing of younger and older populations.

## Introduction

The incidence and prevalence of various mental and physical chronic diseases are increasing globally due to the ageing population. The National Alliance on Mental Illness states that in the year 2020, 21% of the US adult population were diagnosed with mental illnesses. Individuals living with mental health disorders are almost twice as likely to develop serious physical illnesses.^1^ For example, people diagnosed with depression have a 40% higher risk of developing cardiovascular and metabolic complications when compared with the general healthy population.^2^ In addition, some mental disorders may lead to suicide, which is the second-leading cause of mortality among people aged 10–34 years.^2^

Currently, over 46 million people worldwide are living with dementia, and this number is predicted to double every 20 years. Dementia is a clinical syndrome characterized by progressive cognitive deterioration and inability for the individual to live independently.^3^ The prevalence of dementia is greater among older individuals compared with younger ones. Nevertheless, it is crucial to acknowledge that the impact of dementia is not confined exclusively to this demographic; cases can manifest in individuals across a wide range of age groups.^4^ Dementia is a leading contributor to the development of disabilities. The ageing population has led to the global emergence of the dementia epidemic, and governments, especially in low- to middle-income countries, are struggling to respond abruptly and appropriately to the situation. Currently, there is no cure for dementia, making it vital to find easy and cost-effective methods to tackle the dementia epidemic.^3^

The World Health Organization estimates that between 2015 and 2050, the percentage of the global population over 60 years of age will double from 12% to 22%, increasing from 1.4 to 2.1 billion people.^2^ The changing demographics towards an ageing population pose challenges to the healthcare system, families, governments, private-sector organisations, and the individuals themselves. Ageing is biologically associated with age-related health outcomes, as individuals experience a decrease in physical and mental capacity, and an increased risk of developing diseases. These diseases can include osteoarthritis, chronic obstructive pulmonary disease, diabetes, depression, dementia, and cancer.^2^ Specifically, cancer is the second-leading cause of death in the United States and the leading cause in many European countries. Its aggressive treatment can result in long-term psychological damage to the patients, making it vital to develop mechanisms to support their psychological wellbeing.^5^

Older individuals aged >65 years have a higher rate and duration of hospitalisation.^2^ Often, the long duration of hospitalisation can cause patients to feel socially isolated, which can further affect them physically and psychologically.^6^ Chronic loneliness is associated with worsened physical and mental health and increases the risk of mortality.^7^ The number of individuals who feel lonely increased significantly following the COVID-19 pandemic. The outbreak caused lockdowns in which social distancing had to be maintained for prolonged periods, thereby causing an increase in the rates of reported loneliness and increased prevalence of mood disorders, self-harm, suicide and amplification of pre-existing mental health conditions. Therefore, studies suggest that without adequate intervention, loneliness can negatively impact the physical health and psychological wellbeing of an individual.^8^

Several studies have provided evidence for the role of museum and art gallery-based heritage activities and programmes in the prevention and management of disease, and promotion of physical and mental wellbeing. For example, evidence from the literature suggests that art therapy encourages cancer patients to find creative ways to deal with any pain or misfortune their diagnosis has brought and helps strengthen their ability to be goal-orientated, be satisfied, and improve their personal growth.^9^

In a cohort study, researchers investigated the association between attendance at cultural events, including visits to the museums and cancer-related mortality.^10^ A randomly selected group of Swedish adults who were initially cancer-free were followed from 1990 until 2003. The results of study provided evidence that moderate attendees of cultural events had an increased likelihood of dying from cancer during the follow-up period compared with frequent attendees. However, it is important to note that this was only observed in residents of urban areas. Therefore, the results of the study indicated that promoting attendance at cultural events may help improve the health of urban populations.^10^

In one study, Chatterjee et al.^11^ assessed the impact of handling museum objects on patient wellbeing. Using qualitative comparative analysis from self-evaluated reports, the study showed that there was an increase in life satisfaction and health status post intervention. The study provided evidence that museum object handling can help counsel patients, particularly older patients, about issues of illness, death, loss, mourning, while helping restore their dignity, respect and sense of identity. Therefore, the wellbeing and mental health of the older individuals can benefit from the museum interventions involving object handling.^11^

Given the increased prevalence of mental and physical chronic diseases, the changing populations towards an ageing population, and the increased healthcare costs, governments are under pressure to find innovative, effective and easily accessible methods for preventing and not just managing chronic mental and physical diseases, as their impact is detrimental to societies. In recent years, efforts have been made to integrate museums into patients’ lifestyles due to their value in offering non-pharmacological therapeutic methods to heal and improve the patient’s experience with the disease. This aligns with the most recent developments in the heritage sector and the re-evaluation of the role of the museum. The International Council of Museums’ resolution (ICOM) highlights the contribution of museums to sustainable development as an essential element of their agenda; museums should tackle diverse and challenging areas such as populism and ageing societies, focusing on themes like inclusion, health and wellbeing.^12^

An interesting 2021 cross-disciplinary literature review aimed to investigate if and how culture has been linked with public health reports that further studies should be conducted to derive solid evidence on this association.^13^ Meanwhile, museums are positively contributing to the improvement of health by implementing specific and creative programmes to increase patient participation, spreading awareness, and improving social interactions. The Museum of Modern Art in the United States created the first art gallery-based programme targeted for dementia audience,^14^ and London’s Dulwich Picture Gallery in Europe created an art programme targeted at older people, which consists of an ‘art on prescription’ programme with local physicians.^15^ Hence, through their promotion of cultural participation, museums and art galleries can enhance the health and wellbeing of individuals.^16^ Museums are generally considered as safe spaces for visitors, creating an environment that can serve therapeutic purposes.^17^

The aim of this review is to explore studies that have investigated the effect of museums and art gallery-based heritage activities and programmes on the wellbeing of (1) individuals recovering from addiction and individuals experiencing mental health issues or dementia and (2) younger and older populations.

## Methods

### Databases

In the current review, we conducted a search using the following databases: PubMed, EBSCOhost, ProQuest Research Library, Google Scholar, ScienceDirect, DynaMed, ClinicalKey, AccessMedicine, Web of Science, UpToDate, Scopus, Scival, BrowZine, Wiley and LiBKey.

### Search strategy

The search strategy applied the following keywords with the appropriate application of Boolean operators:

- ‘Museums’ AND (‘wellbeing’ OR ‘health’ OR ‘mental health’).
- (‘Art gallery’ OR ‘Participatory arts’ OR ‘creative art’ OR ‘creative activity’) AND (‘wellbeing’ OR ‘health’ OR ‘mental health’).

### Inclusion and exclusion criteria

#### Inclusion criteria

We included original research studies (observational, interventional, cohort studies) that explored the impact of museum or art gallery-based heritage activities and programmes (defined as heritage activities or programmes conducted in a physical museum or art gallery) on the wellbeing of (1) individuals recovering from drug addiction or patients with dementia and (2) younger and older populations. We only included studies, in which the physical location of the activity or programme conducted was either a museum or an art gallery, that were published during the period of 2013–2023, and were written in the English language.

#### Exclusion criteria

We excluded secondary research journal articles (narrative, scoping or systematic reviews) and original studies that (1) were not relevant to the topic of the review article (as described above), (2) included participants with other chronic diseases, (3) were not conducted in museums or art galleries, (4) were published before the year 2013 and (5) were not written in the English language.

Following a detailed examination of numerous articles, 15 original studies were included in the current review article.^18^

## Results

The 15 articles were sub-categorised into research studies that investigated whether museum and art gallery-based heritage activities or programmes improved the wellbeing of:

1. Individuals recovering from addiction recovery participants, patients with mental health diagnosis and patients with dementia.^18–25^
2. Younger and older adults.^26–32^

### Museum and art gallery-based activities or programmes in addiction recovery and dementia

In this article, we have included eight studies that investigated the impact of museum art-based programmes on individuals recovering from addiction and dementia patients (Table 1).^18–25^

**Table 1.** Primary research studies investigating the implementation of museum or art gallery-based activities or programmes on the wellbeing of individuals recovering from addiction, individuals with mental health challenges and patients with dementia

Reference	Type of study	Study aim	Participants, method and data analysis	Study outcomes	Main conclusions
Study with individuals recovering from addiction and individuals with mental health challenges					
Morse et al.^18^	Museum-based interventional study including quantitative and quantitative analysis	• To examine the effects of museum outreach sessions on confidence, sociability and wellbeing measures for mental health and addiction recovery service-users.	Participants: • Mental health participants (*n* = 85) and addiction recovery participants (*n* = 59) Method: • Both groups participated in weekly outreach sessions combining object handling and museum visits with arts and craft activities. Data analysis: • Using mixed methods, measures of confidence, sociability and wellbeing were evaluated quantitatively through a ‘ladder of change’ model of steps towards independence and feedback was analysed qualitatively.	• Comparison of scores of participants from first, mid- and last sessions showed increases across all measures (confidence, sociability and wellbeing). • Qualitative analysis revealed additional gains including pride, learning and skills, and creativity. Findings were interpreted in terms of social capital, independence and resilience.	• Creative museum activities may increase the participants’ levels of confidence, sociability and wellbeing. • Future research should investigate the value of museum activities to health and wellbeing.
Studies with dementia patients					
Camic et al.^19^	Art gallery-based interventional study mixed-methods design	• To examine the impact of an 8-week art gallery-based intervention on people living with mild to moderate dementia and their carers.	Participants: • n = 24 participants (12 dementia, 12 carers). Method: • 8-week art gallery-based intervention Data analysis: • Qualitative analysis (thematic analysis). • Mixed-methods pre–post design using interviews and questionnaires to examine social inclusion, carer burden, quality of life, and daily living activities for a person with dementia.	• No significant pre–post difference was absorbed. • Slight decrease in burden for the carers of dementia patients was noted. • Thematic analysis showed that following the completion of the programme, patients felt more socially included and had improved cognitive capacities with improved quality of life.	• Art-based gallery interventions help foster social inclusion and engagement, stimulate cognitive processes of attention and concentration, and enhance the relationship between dementia patients and their carers.
Camic et al.^20^	Art gallery-based interventional study with qualitative analysis	• To understand the impact of an art gallery Intervention on dementia patients and their caregivers.	Participants: • Patients with mild-to-moderate dementia (*n* = 12) and their caregivers (*n* = 12). Method: • Participants engaged in viewing and creating art for a period of 8 weeks. Data analysis: • Data were collected via post intervention interviews which were then analysed using grounded theory methodology.	• The art gallery provided those with dementia with intellectual stimulation and opportunities for social inclusion, which created a positive emotional and relational effect.	• The study suggests the use of art gallery-based programmes in the care of patients experiencing dementia within the healthcare setting.
Johnson et al.^21^	Museum-based quasi-experimental crossover design study with mixed design analysis	• To investigate the impact of two museum-based activities and a social activity on the subjective wellbeing of people with dementia and their caregivers.	Participants: • People with early to middle stage dementia (*n* = 36) and their caregivers (*n* = 30). Method: • Participants took part in museum object handling, a refreshment break and art viewing in small groups. Data analysis: • Visual analogue scales were used to rate subjective wellbeing pre and post each activity.	• Mixed-design analysis of variances indicated wellbeing increased from object handling and art viewing for those with dementia and caregivers across pooled orders, but did not increase in the social activity of a refreshment break. • An end-of-intervention questionnaire indicated that experiences of the session were positive.	• Museum activities should be considered as part of a broader psychosocial, relational approach to dementia care and support the use of easy to administer visual analogue scales as a quantitative outcome measure. • Museums and healthcare professionals should work together for the development of non-clinical, community-based programmes for this population.
Schall et al.^22^	Museum-based interventional study including quantitative analysis	• To investigate if an art-based interventional study can improve the quality of care for patients with dementia through museum visits and artistic activity.	Participants: • Patients with mild-to-moderate dementia (*n* = 44). Method: • ‘ARTEMIS’ (ART Encounters: Museum Intervention Study) • Patients and their care partners visited the Frankfurt Stadel Museum once a week, for a total period of 6 weeks. • Each intervention consisted of a 1-h guided art tour followed by an hour of art-making in the studio session. Data analysis: • The mixed-methods design assessed cognitive status, emotional wellbeing, self-rated aspects of quality of life, and subjective evaluations by the patients’ caretakers.	• A comparison of pre- and post-assessment showed that the intervention significantly improved the participants’ self-rated quality of life. • The Neuropsychiatric Inventory score as well as the affective and apathy subscales were significantly reduced compared with baseline following the ARTEMIS intervention.	• Museum-based art interventions can improve the subjective wellbeing, mood and quality of life of patients with dementia.
Camic et al.^23^	Museum-based Quasi-experimental design	• To examine the effect of museum object handling on patients with dementia considering their differences across domain, time, gender and stage of the disease.	Participants: • Patients (*n* = 80), aged 54–89 years, with mild-to-moderate levels of dementia under 5 years of diagnosis. • 50 of the patients had early-stage dementia and 30 had moderate-stage dementia. • The diagnoses were Alzheimer’s (*n* = 37), vascular (*n* = 24), frontotemporal (*n* = 4), mixed-types (*n* = 13) and HIV-related (*n* = 2). Method: • The participants took part in sessions which involved handling museum artefacts following a quasi-experimental design. Data analysis: • Differences between the two groups were examined for domain, time, gender and stages of dementia.	• Patients experiencing early to moderate stages of dementia had a more positive increase in wellbeing in comparison to the rest of the subgroups.	• Museum object handling can have a positive impact on the wellbeing of patients with early and moderate-stage dementia. • Art-based interventions could be implemented in the management of patients with dementia.
D’Cunha et al.^24^	Art gallery-based intervention, quasi-experimental design	• To investigate the physiological outcomes of patients with dementia following the attendance of an art gallery-based programme.	Participants: • People with dementia (*n* = 28) Method: • Participants joined a six-week programme and were followed for 12 weeks. Data analysis: • The study measured the levels of salivary biomarkers of cortisol and interleukin-6 (IL-6), quality of life (QoL), depressive symptoms, cognition, and wellbeing pre- and post-intervention.	• Waking to evening salivary cortisol ratio was higher post intervention, but had returned to baseline during the follow-up period. • No change in interleukin-6 levels or quality of life were observed. • Self-reported depressive symptoms decreased and memory and verbal fluency improved in post intervention compared with the baseline.	• The NGA Art and Dementia programme improves hypothalamic–pituitary–adrenal axis function, a controlled trial of longer duration is recommended to evaluate the physiological impacts of the programme on dementia patients.
Bourne et al.^25^	Art gallery-based intervention, quasi-experimental design	• To investigate the physiological and psychological outcomes of patients with dementia following the attendance and participation in visual arts activities.	Participants: • People with dementia in the mild to moderate stages (*n* = 1109) Method: • Participants were asked to participate in 60 min of singing or 2 h of art viewing Data analysis: • The study measured physiological measures of salivary cortisol, heart rate, and heart rate variability, and their psychological measures which included subjective stress and wellbeing once before and after the participation.	• The objective physiological results indicated non-significant decrease in the saliva levels and a non-significant increase in stress levels for the patients with dementia in comparison to baseline • The quantitative results showed that the optimism and happiness scores for patients with dementia significantly increased from baseline in choral group. • The majority of participants reported non-significant decrease in stress following the session.	• The subjective psychological outcomes for patients with dementia significantly improved following participation in visual arts activities. • Further investigations are required to re-assess the factors.

#### Study on individuals recovering from addiction and individuals with mental health challenges

One study was performed with individuals recovering from addiction (Table 1).^18^ The art-based interventional study by Morse et al.^18^ investigated whether museums improved the confidence, sociability, and wellbeing of individuals recovering from addiction and individuals experiencing mental health challenges. In this study, both groups were involved in weekly sessions that combined museum encounters with hands on arts and crafts activities. The results of the study showed that the specific creative museum activities increased the participants’ confidence, sociability, and wellbeing.

#### Studies with dementia patients

Seven studies investigated the effect of museum art-based programmes on patients experiencing dementia (Table 1).^19–25^

One study by Camic et al.^19^ examined the impact of an 8-week art gallery-based programme on people living with mild to moderate dementia and their carers. The study measured changes in social inclusion, carer burden, quality of life and daily living activities for persons with dementia pre- and post-intervention. Although no significant pre–post difference was observed, a slight decrease in the burden experienced by carers of dementia patients was noted. In addition, patients who participated in the programme felt more socially included and had enhanced cognitive capacities and improved quality of life. Therefore, future studies should investigate if museum and art gallery-based heritage activities or programmes can help foster social inclusion and engagement, stimulate cognitive processes of attention and concentration, and enhance the relationship between the dementia patients and their carers.^19^

In another study by the same group, Camic et al.^20^ explored the effect of art gallery-based interventions on dementia patients and their caregivers. The participants engaged in viewing and creating art for a period of 8-weeks. Data were collected via post intervention interviews, which were subsequently analysed using grounded theory methodology. The results reported that the art gallery-based programme provided individuals experiencing dementia with intellectual stimulation and opportunities for social inclusion, creating a positive emotional and relational effect.^20^

The role of museum programmes on the quality of life of early stage dementia patients was also examined by another study by Johnson et al.^21^ In this study, patients with early to middle-stage dementia and their caregivers participated in museum object handling, a refreshment break and art viewing in small groups. The results showed that object handling and art viewing increased the wellbeing of people with dementia and their caregivers.^21^

Schall et al.^22^ developed an interesting art-based interventional study which explored whether museum visits and artistic activity could improve the quality of care for patients with dementia. In this study, participants with mild to moderate dementia and their care partners visited the museum once a week, for a period of 6 weeks. Each intervention consisted of a 1-h guided art tour followed by an hour of art-making in the studio session. Comparison of pre-assessment to post assessment provided evidence that the intervention significantly improved the participants’ self-rated quality of life. The researchers concluded that museum-based art interventions can improve the subjective wellbeing, mood and quality of life of patients with dementia.^22^

The role of art-based interventions on improving the quality of life of patients diagnosed with dementia was also investigated in a study by Camic et al.,^23^ who examined the effect of museum object handling on patients with dementia. Patients with mild-to-moderate levels of dementia participated in sessions which involved handling museum artefacts. The results of the study demonstrated that for patients with early to moderate-stage dementia, museum object handling had a positive impact on their wellbeing and that similar art-based interventions could be implemented in the care of patients with dementia.^23^

D’Cunha et al.^24^ investigated the physiological outcomes of patients with dementia following the attendance of a 6-week art gallery-based programme. The study measured the levels of salivary biomarkers of cortisol and interleukin-6 (IL-6), quality of life, depressive symptoms, cognition and wellbeing pre- and post-intervention. The results demonstrated that the waking to evening salivary cortisol ratio was higher post intervention, but these levels returned to baseline in the following months. In addition, no change in IL-6 levels or quality of life was evident. On the contrary, self-reported depression decreased post intervention compared with baseline, with an improvement in memory and verbal fluency. Therefore, the programme improved hypothalamic–pituitary–adrenal axis function, but there is a need for follow-up research such as the implementation of a controlled trial of longer duration, to evaluate the physiological effect of the programme on dementia patients.^24^

Bourne et al.^25^ assessed the role of the visual arts activities in 1109 dementia patients in their mild to moderate stages of the disease by measuring their physiological and psychological responses. The patients were divided into two categories, choral singing and/or art viewing, and then compared with their baseline physiological measures of salivary cortisol, heart rate and heart rate variability, and their psychological measures which included subjective stress and wellbeing. Their caregivers were also studied in the experiment as controls. Following singing for 60 min and art viewing for 2 h, the psychological and physiological factors were measured and compared in a quasi-experimental design. The objective physiological results indicated a non-significant decrease in saliva levels and a non-significant increase in stress levels for the patients with dementia, although the subjective quantitative results showed that the optimism and happiness scores for patients with dementia significantly increased from baseline in the choral group, perhaps because they were emotionally engaged more than during the art-viewing session. The majority noted a non-significant decrease in stress following the session.^25^

### Museum and art gallery-based heritage programmes and the wellbeing of young and older individuals

Seven studies investigated the effect of museum and art gallery-based heritage activities and programmes on the wellbeing of the general population (young and elderly) (Table 2).^26–32^

**Table 2.** Primary studies investigating the role of museum or art gallery-based activities or programmes on the wellbeing of younger and older populations.

Reference	Type of study	Study aim	Participants, method and data analysis	Study outcomes	Main conclusions
Young populations					
Kanhadilok and Watts^26^	Art-based intervention with qualitative and quantitative analysis	To explore the playfulness of young people in a museum setting.	Participants: • 60 children Method: • Children visiting the Thai Toy gallery of the National Science Museum in Thailand were selected and their social interactions were examined using Bandura’s social learning theories and Lieberman’s categories to measure playfulness. Following the intervention, the adults accompanying their children were asked to fill out a questionnaire n describing their children’s character. Data analysis: • Qualitative and quantitative analysis of results of observations and questionnaire surveys.	• The Youth adults enjoyed the activities, and were reported to be more happy, funny and cheerful. Only a small percentage were distant and unengaged in the activities.	• Play-based learning can be a significant design element for informal educational places such as museums.
Mastandrea et al.^27^	Art-based intervention and quantitative analysis	• To assess, through physiological measurements whether exposure to art museums and to different art styles (figurative vs. modern art) can enhance visitors’ wellbeing.	Participants: • Undergraduate students (*n* = 77, female = 64, mean age = 22.5 SD = 5.2). Method: • Participants were randomly assigned to one of three groups on the basis of the typology of the art style they were exposed to in the museum visit: (1) figurative art, (2) modern art and (3) museum office (as a control condition). Blood pressure and heart rate were measured before and after the visits. Data analysis: • Analys is of blood pressure and heart rates of participants pre- and post-visits at the museum.	• The majority of the participants exposed to figurative art significantly decreased systolic blood pressure compared with those exposed to modern art and museum office.	• Museum visits can have health benefits, and figurative art may decrease systolic blood pressure.
Batat^28^	Art-based intervention with qualitative analysis	• To better understand what a younger visitor’s daily environment is and how museums can be developed according to their needs by studying their behaviour.	Participants: • 32 young adults, aged from 13 to 18 years. Method: • In-depth interviews were conducted, focusing on gathering information on the adolescent’ experiences and perceptions of art museums and exhibitions. Data analysis: • Qualitative analysis of interviews.	• Adolescents are more likely to spend their leisure time doing other things, and perceive visiting museums as a task that is done only if required.	• Museums need to be more youth-centred for their businesses to grow and for the youth to gain academic knowledge.
Lacoe et al.^29^	Art-based intervention & Cross-sectional study	• To identify if the participation of low-income students in museum-based intervention can have any short- and long-term academic and behavioural outcomes.	Method: • Longitudinal, student-level data was used from 1996 from one elementary school with programme implementation titled School in the Park (SITP) (a museum-based educational programme for low-income students that took place within the cultural institutions and museums of San Diego’s Balboa Park) and the other elementary school that did not have access to the programme (the control group). Data analysis: • The impact of participation in SITP on participating students was measured and analysed using descriptive statistics.	• Students participating in SITP had small short-term improvements such as increase in test-scores, and decrease in suspensions and detention. • An increase in the number of weeks participating in SITP positively correlated to an increase in the positive effects. • Participation in the SITP programme had positive short-term effects which were insignificant in the long-term.	• Further research needs to be conducted to examine if the participation of low-income students in art-based intervention programmes can have any short- and long-term academic and behavioural outcomes.
Older populations					
Todd et al.^30^	Art-based intervention and qualitative analysis	• To understand how museums, enhance wellbeing and health, as well as reducing the feeling of social isolation in older adults	Participants: • 20 participants who self-identified as socially isolated were sampled from a study of 115 people (aged 65–94) Method: • Participants participated in a session of two-hours per week over 10 weeks (in one of seven museums in central London and Kent). Measures of wellbeing and social isolation were recorded pre-study, and throughout the programme. • Post programme interviews (45–90 min) were conducted followed by further interviews at 3 month follow-up (20–30 min). Data analysis: • Analysis of post programme interviews and quantitative analysis of questionnaires.	• The study reported that museums provide opportunities for increasing and creating more intense social experiences, as well as having a positive influence on emotion, health, activity levels, expectations and how participants present themselves.	• To better enhance wellbeing and support socially isolated older people, museums need to be easily accessible, engaging, and support social interactions by running several sessions daily, and be facilitated by well-informed staff.
Thomson et al.^31^	Art-based intervention with qualitative and quantitative analysis	• To investigate if the wellbeing of older attendees can benefit from programmes offered at museums.	Participants: • Participants (*n* = 115) aged 65–94 were selected using inclusion criteria (socially isolated, able to provide informed consent, not in employment, and do not regularly attending social or cultural activities) and exclusion criteria (unlikely to attend all sessions, or complete questionnaires and interviews). Method: • Participants were referred to attend museum-based programmes comprising of 10 weekly museum sessions. • The Museum Wellbeing Measure for Older Adults (MWM-OA) was administered pre–post session at start-, mid- and end-programme (information was collected on six self-rated MWM-OA emotions – (‘absorbed’, ‘active’, ‘cheerful’, ‘encouraged’, ‘enlightened’ and ‘inspired’). • Participants, carers (where present), museum staff and researchers kept weekly diaries following guideline questions and took part in end-programme in-depth interviews. Data analysis: • qualitative analysis of interviews and quantitative analysis of questionnaires.	• The results of the study were analysed using multivariate analysis and demonstrated significant improvements of the participants in all six MWM-OA emotions.	• Museums can be important in offering programmes for older adults and may improve their psychological wellbeing over time.
Beauchet et al.^32^	Art-based intervention with randomized, clinical controlled trial	• To assess the participation of older community dwellers in “Thursdays at the Museum” (intervention group) on changes in wellbeing, quality life, frailty, and physiological measurements.	Participants: • 150 older community dwellers (*n* = 75 in intervention group, *n* = 75 in control group). Method: • The intervention group attended “Thursdays at Museum,” a 2 hour art-based workshop, for a period of 3 months. The control group did not take part in any art-based activities. • The assessments of the measurements took place at baseline, beginning of the 2nd and 3rd months, at the end of the third month, and after 6 and 12 months of the last workshop. Data analysis: • Quantitative analysis of interviews measuring primary and secondary outcomes.	• A decrease in frailty score, improvement in wellbeing and quality of life as seen by an increase in Warwick-Edinburgh Mental Wellbeing Scale (WEMWBS) and EuroQol-5D (EQ-5D) were reported in the intervention group post intervention compared with pre-intervention.	• Museums can play a key role in the wellbeing and health promotion of the elderly. • MMFA participatory art-based activities can have a positive impact on mental and physical health and could be implemented to improve the wellbeing of elderly individuals.

#### Studies with young populations

Four studies investigated the effect of museum and art gallery-based heritage activities or programmes on the wellbeing of young populations (Table 2).^26–29^

Kanhadilok and Watts^26^ explored the playfulness of young people in a museum setting.^28^ The results of this study showed that young adults enjoyed participating in the activities and were reported to be more happy, funny and cheerful, whereas only a small percentage were distant and unengaged in the activities. The study concluded that play-based learning can be a significant design element for informal educational places such as museums.^26^

A recent study by Mastandrea et al.^27^ assessed whether exposure of young undergraduate students to art museums and to different art styles (figurative vs. modern art) could enhance their wellbeing and reduce their stress. The results showed that even though the diastolic values of the participants remained stable, the majority of participants exposed to figurative art had significantly decreased systolic blood pressure compared with participants exposed to modern art. Therefore, the findings of this study indicate that museum visits involving exposure to figurative art can have health benefits for young individuals and further research should investigate this further.^27^

A recent study by Batat^28^ explored the role museums may play for younger visitors by studying their behaviour and understanding their daily environment. By conducting in-depth interviews with young adults, the researchers derived data on the adolescents’ experiences and perceptions of art museums and exhibitions. The study reported that most adolescents were not interested in museums and did not share the same perception as professionals for cultural tourism. Cultural tourism, defined as travelling with a purpose of gaining knowledge through experiencing cultural contexts, customs, traditions, local lifestyle, landscapes, arts and so on, is an important characteristic for adolescents to acquire experience and knowledge of their surrounding environment. Therefore, the results of the study indicated that museums need to be more youth-centred not only for their businesses to grow but also to help achieve their educational purpose and assist the youth in increasing their academic knowledge.^28^

Lacoe et al.^29^ examined the effect of low-income student participation in the ‘School in the Park (SITP)’ programme on short- and long-term academic and behavioural outcomes. Longitudinal, student-level data were used from one elementary school with programme implementation titled ‘School in the Park’, and another elementary school that did not have access to the programme – the control group. Students participating in SITP had small short-term improvements such as increase in test scores and decrease in suspensions and detentions. In addition, an increase in the number of weeks participating in SITP positively correlated with the increase in positive outcomes. Overall, participation in the programme had positive short-term effects, which were, however, insignificant in the long-term.^29^ Therefore, further research needs to be conducted to examine if the participation of low-income students in art-based intervention programmes can have any short- and long-term academic and behavioural outcomes.

#### Studies with older adults

Three studies were conducted to assess the effect of museum or art gallery-based heritage activities or programmes on the wellbeing of the older adults (Table 2).^30–32^

Todd et al.^30^ investigated how museums enhance wellbeing and health as well as reduce the feeling of social isolation in older adults. Participants who self-identified as socially isolated were sampled from older participants who were interviewed after participating in programmes across seven museums in the UK. The wellbeing and social isolation of the participants were recorded prior to the study and throughout the programme, which consisted of 2-h visits for a period of 10 weeks. The study reported that museums provide opportunities for increasing and creating more intense social experiences and have a positive influence on emotion, health, activity levels, expectations and how participants present themselves. To better enhance wellbeing and support socially isolated older people, museums need to be easily accessible, engaging and supportive of social interactions by running several sessions daily and must be facilitated by well-informed staff.^30^

In the study by Thomson et al.^31^ older participants selected using specific inclusion criteria (socially isolated, unemployed and not regular attendees of social or cultural activities) were referred to attend museum-based programmes comprising 10 weekly museum sessions. The Museum Wellbeing Measure for Older Adults (MWM-OA) was performed at pre- and post-sessions, at start-, mid- and end-programme. Moreover, participants (including their carers), museum staff and researchers were requested to keep a record in their weekly diaries and participated in end-programme interviews. The results demonstrated significant improvements of the participants in six MWM-OA emotions which included ‘absorbed’, ‘active’, ‘cheerful’, ‘encouraged’, ‘enlightened’ and ‘inspired’. Therefore, museums can play an important role in offering programmes for older adults and may eventually enhance their psychological wellbeing.^31^

Finally, one study by Beauchet et al.^32^ recruited older community dwellers who participated in an intervention titled ‘Thursdays at the Museum’ and assessed their wellbeing, quality life, frailty and physiological measurements. The intervention group attended a 2-h programme every Thursday for a period of 3 months, while the control group did not participate in any art-based activities. The results reported an improvement in wellbeing and quality of life and a decrease in the frailty score in the intervention group in comparison to no change in the control. The results provide evidence for the important role museums can play in wellbeing and health promotion. Therefore, participatory art-based activities have a positive impact on mental and physical health, which can support the implementation of these programmes in the lifestyle of older populations.^32^

## Conclusion

In the current review, we explored the effect of museum or art gallery-based heritage activities or programmes on individuals experiencing mental health challenges or recovering from addiction or dementia patients and the general population, focusing on the youth and older adults.

Museum and art gallery-based heritage activities and programmes have been gaining the attention of many researchers due to increasing evidence from the literature supporting their use as a non-pharmacological tool for improving wellbeing. Insights from these studies suggest that for individuals recovering from addiction or experiencing mental health challenges, such programmes may increase levels of confidence, sociability and wellbeing. For dementia patients, these programmes may foster social inclusion and engagement, stimulate cognitive processes of attention and concentration, enhance the relationship between patients and their carers, improve mood and increase the quality of life and wellbeing. For the young population, the studies provided evidence of positive effects, yet museums should be more youth-centred. Studies conducted with older individuals provided evidence that these programmes may improve their psychological wellbeing over time; yet museums need to be easily accessible, engaging and support social interactions.

The studies identified in the current review have provided some valuable contributions to our understanding, but the overall literature in the field suffers from several limitations. These limitations include the low number of participants, short duration of each study and the heterogeneous methodology applied by each study, making it difficult to derive statistically conclusive results. Therefore, further studies are necessary to draw more definitive conclusions on the impact of museum and art gallery-based heritage activities and programmes on the wellbeing of patients and the general population.

While considering the development of museum and art gallery-based programmes, it is essential to address the limitations identified in previous studies, which include small participant numbers, short duration and variable outcomes across different studies. For future endeavours a recommended approach involves adopting a quasi-experimental mixed methodology (an approach involving both quantitative and qualitative methods). However, it is crucial to acknowledge the inherent challenges associated with designing and implementing randomization for non-clinical interventions. To address these problems a potential solution is to employ larger sample sizes within a longitudinal quasi-experimental framework. This may involve implementing a waiting list control structure which can enhance the robustness of the study. In addition, incorporating more qualitative research methods is advisable. Qualitative methods provide a valuable means to capture the participants’ perspective and voice, shedding light on the lived and felt experiences associated with the implementation of the specific programmes.

Collecting information via qualitative methods is particularly beneficial, as it may allow the extraction of information that cannot be collected via quantitative research. Having a more in-depth understanding of the participants’ experiences, feelings, barriers and facilitators can contribute significantly to the evaluation of these programmes. In summary, the combination of larger sizes, a longitudinal approach and the application of qualitative methods will help the collection of valuable information for the evaluation of such programmes and the development of future programmes. Therefore, it is recommended for museums and art galleries to work with multiprofessional teams to collaborate together in the design, implementation and assessment of programmes that can promote wellbeing in participants facing challenges with their mental health, have been diagnosed with a chronic disease, or are part of the general population. It is implied that different population groups may have specific needs and may require the development of different programmes to cater for their specific needs and characteristics.

In summary, museum and art gallery-based activities or programmes should be considered as part of a broader psychosocial, relational approach to the improvement of wellbeing. Such approaches could involve patients diagnosed with chronic disorders and should be investigated to see if they can support patients in finding creative ways to deal with any pain or misfortune their diagnosis has brought, while helping to strengthen their ability to be goal-orientated, satisfied and improve their personal growth. These activities can also be implemented for socially excluded or vulnerable individuals. Most importantly, museum-based interventions could be used for the promotion of overall wellbeing across entire populations, adjusting to the specific needs of different age groups.
